# Children with autism spectrum disorder show atypical electroencephalographic response to processing contextual incongruencies

**DOI:** 10.1038/s41598-022-12475-z

**Published:** 2022-05-27

**Authors:** Amparo V. Márquez-García, Vasily A. Vakorin, Nataliia Kozhemiako, Justine R. Magnuson, Grace Iarocci, Urs Ribary, Sylvain Moreno, Sam M. Doesburg

**Affiliations:** 1 Department of Biomedical Physiology and Kinesiology, Simon Fraser University, Burnaby, Canada; 2 Biomedical Physiology and Kinesiology, Behavioral & Cognitive Neuroscience Institute, Simon Fraser University, Burnaby, Canada; 3 Department of Psychology, Simon Fraser University, Burnaby, Canada; 4 Department of School of Interactive Arts & Technology, Simon Fraser University, Burnaby, Canada

**Keywords:** Neuroscience, Biomarkers

## Abstract

Children with autism spectrum disorder (ASD) experience difficulties with social communication, making it challenging to interpret contextual information that aids in accurately interpreting language. To investigate how the brain processes the contextual information and how this is different in ASD, we compared event-related potentials (ERPs) in response to processing visual and auditory congruent and incongruent information. Two groups of children participated in the study: 37 typically developing children and 15 children with ASD (age range = 6 to 12). We applied a language task involving auditory sentences describing congruent or incongruent images. We investigated two ERP components associated with language processing: the N400 and P600. Our results showed how children with ASD present significant differences in their neural responses in comparison with the TD group, even when their reaction times and correct trials are not significantly different from the TD group.

## Introduction

Difficulty understanding a joke or knowing whether a compliment is genuine or sarcastic or whether someone is upset or sad can result in a social misunderstanding at best and, at worst, social injustices depending on the circumstances. To understand our social environment, we need to integrate and interpret information within its broader context. According to the weak central coherence theory proposed by Uta Frith in 1989^1,2^, individuals with autism spectrum disorder (ASD) experience difficulties integrating contextual information for global meaning, which is critical for making sense of the world^1^. ASD is a developmental disorder defined by difficulties in social communication and restricted/repetitive interests and behaviours^3^.

One aspect of communication that may be impacted in an individual with ASD is pragmatic language competence, which is defined as the ability to interpret language in context^4–7^. It may be challenging for individuals with ASD to participate in conversations due to their difficulties interpreting nonverbal aspects of language and language in context. Difficulties integrating specific information can result in individuals with ASD not wholly understanding the people and the world around them.

The interpretation of language in context is a challenge even for children with ASD who are fluently verbal^8,9^, that may persist into adulthood^9,10^. In addition, language difficulties in ASD have been robustly established at the behavioural level^6,11–22^. The neural mechanisms underlying these language difficulties, however, remain poorly understood. Such mechanisms can be explored with electroencephalography (EEG), a non-invasive and direct measure of neural activity. Event-related potentials (ERPs) detected with EEG can measure neural correlations of language processing and multisensory integration in ASD^23,24^. ERP reflects the time-locked electrophysiological responses to stimuli. ERPs measure the synchronized post-synaptic activity of large groups of neurons, reflecting sensory and cognitive processing in response to a stimulus presentation. ERP components are classified according to their polarity (positive or negative deflection) and peak latency.

The high temporal resolution of ERPs enables tracking of language processing with millisecond resolution^25^. Accordingly, this approach is well suited for evaluating the incremental nature of language^26^. Two ERP components have been consistently linked to sentence comprehension in electrophysiological investigations of language processing, the N400 and the P600. The N400 is a negative potential peak at 400 ms after word onset, while the P600 is a positivity most pronounced 600–900 ms after word onset^27,28^. The N400 is the most widely used ERP in language research^27^ and reflects semantic processing. Perception, attention, memory, and language jointly participate in the neural events responsible for the N400^26^.

According to Kutas and Federmeier^26^, the N400 effect is largest over centro-parietal sites, with a slight bias toward the right hemisphere (at least, for written words in sentences). Previous studies had shown an N400 effect elicited in unimodal tasks such as involving spoken words, written words and sentences, pictures, arithmetic tasks and more^26,29–34^. The N400 has also been recorded in cross-modal paradigms, where auditory target words are preceded by visual priming words^35–41^.

Magnetoencephalography (MEG) studies using dipole modeling typically identify the sources of N400 in the left superior and/or middle temporal gyri, with a homologous source in the right hemisphere region, which is considered more variable^42–46^. Using a distributed source modeling, Halgren et al.^42^, showed the spatial extent of the cortical activity when the N400 was elicited. They found that most of the left temporal lobe (including inferior and anterior regions) was more active for incongruent than congruent sentence completions, with additional activity in the right anterior temporal lobe.

N400s can be observed for both visual and auditory words, and thus can be applied to compare across modalities, which is relatively less tractable for response time studies. The functional similarity of the N400-generating process in the two modalities was theoretically important for psycholinguistics. Auditory N400s tended to begin earlier, last longer, and have a slightly more frontal and less right-biased topography (reviewed in (Kutas &Van Petten, 1988)^47^).

While the N400 is a robust measure in typically developing populations, it is unclear if this is equally true for ASD groups. The ERP literature on language processing in children and adults with ASD is scarce. These studies have yielded mixed findings. A few studies have shown reduced amplitude, prolonged latency, and different topology of the N400 in individuals with ASD^48,49^. The other studies have found the N400 response intact, depending on the task^48,50,51^.

Several studies have demontrated that children with ASD failed to show any ERP evidence of semantic processing. Dunn and Bates^52^ used an inverted-out-of-category words task (not a priming task) and found that N400 responses in the ASD group did not differ across conditions. Similarly, McCleery et al. ^53^ compared verbal and nonverbal semantic integration in the high functioning children with ASD. They found that the N400 effect was absent in the children with ASD during a picture-word priming paradigm but was present when the pictures were paired with environmental sounds (i.e., the sound of a car engine starting and ball bouncing).

On the other hand, several studies have found altered but not absent semantic processing in individuals with ASD. Semantic processing was investigated in seven children with high functioning ASD, using a semantic congruence sentence task. They found that there was no N400 response in the children with ASD, but instead, the conditions were differentiated by a late positive component (LPC), which is also known as the P600^54^. Pijnacker et al.^55^ also did not confirm the alterations in N400 responses in adults with ASD, reporting a larger LPC to semantically incongruous sentences. Such mixed findings can be partly explained by the differences in the paradigms used to elicit the N400. The paradigms vary from sensory input (i.e., visual, auditory or multimodal), differences in the complexity of the stimulus (i.e., sounds, words or sentences) and different tasks (i.e., semantic priming task), which makes it difficult to understand the expected behaviour of this ERP component in the ASD population. Nevertheless, these neurophysiological outcomes with complex alterations across space and time suggest that the autistic population may use a different and more elaborate mechanism to understand language in context^55,56^.

The ERP literature has also reported findings on the P600 in the context of language processing. This ERP component is commonly observed in the 500–900 ms time window, with a parietal topography^57^. The P600 component was initially thought to reflect manipulation of syntactic information^57^, but has since been associated with conflict monitoring^58–60^. The P600 response can be found in a wide range of syntactic violations such as phrase structure violation^61^, semantic violations in extended discourse contexts^62^, subject-verb number agreement^63^, pronoun case^47^, verb inflection^64^ and subjacency^65^. The P600 response has been consistently associated with capturing differences between syntactically congruent relative to incongruent syntactic structures (e.g.,^27,66,67^. It has been debated, however, whether the P600 responses seen in these cases are specialized for syntax processing or instead linked to a more general domain process such as attention, context updating or learning^27,47,68,69^. Fitz and Chang ^70^ proposed a model presenting P600 as the prediction error at the sequencing layer of a neural network. Their studies have shown that the recorded ERP components could be the result of learning processes, that helps in the adaptation process to new inputs.

These studies have also suggested that P600 reflects an integration process in the comprehension of the visual world. Sitnikova et al.^71^ presented movie clips of real-world activities with two types of endings: congruent and incongruent with the context. Their results showed that the violations of the expected event elicited the P600 component, which led them to conclude that the comprehension of the visual real-world required the mediation of two mechanisms reflected by N400 and P600. Differences in language processing in ASD individuals are also reflected in the P600 amplitude and latency. When exploring linguistic violations, the group with ASD presented longer reaction times^72^ and broader distributed P600 effects^73^. P600 variations were associated with higher attentional cost and compensatory strategies. However, studies assigning the P600 response exclusively to incongruency in individuals with ASD remain scarce.

Due to the mixed results commonly found in studies of ASD, it is important to identify the paradigms capable of identifying differences in the neural responses to contextual language processing. In our study, we aim to investigate brain processing in children with ASD related to difficulties in the interpretation of language in context. To achieve this, we studied the detection of context incongruencies. We applied a task that demanded integrating visual and auditory information to assess whether a sentence contradicts the context (incongruent condition) or matches the context (congruent condition). The incongruent condition included two different categories: i) incongruent trials with sentences that are grammatically correct, and ii) incongruent trials with sentences that are grammatically incorrect presenting semantic mistakes. We used a 2 × 2 design with images (context) accompanied by an oral description (language) that could be either congruent or incongruent with the image. We examined the ERP waves amplitudes for N400 and P600 components and studied the differences across children with ASD and typically developing controls. We assessed group differences and differences between the two conditions within the groups. We hypothesized that individuals in the typically developing group would detect the incongruencies and, in response, present significantly higher N400 and P600 amplitudes on the incongruent conditions compared to the congruent conditions. We also expected the ASD group to have difficulties detecting the incongruencies between the context and the description. When investigating group differences, we expected to find significant differences in the amplitudes of the N400 and P600 ERPs on the incongruent conditions, with larger ERP amplitudes in the non-autistic group.

## Materials and methods

### Participants

We recruited a population of 75 children, 33 participants with ASD (mean age [years] 9.7 ± 5.6) and 42 typically developing participants (mean age [years] 9.3 ± 2.51). However, only 15 participants with ASD (mean age [years] 10.8 ± 1.4) and 37 typically developing participants (mean age [years] 9.3 ± 1.6) met all of the inclusion criteria. Individuals with an IQ less than 70 and/or with less than 20 correct trials were excluded from the study (Table 1). Participants with ASD had a prior diagnosis of ASD as received by a qualified pediatrician, psychologist or psychiatrist associated with the government-funded ASD assessment network or with a qualified private clinic in British Columbia (BC). The ASD diagnoses were based on the Diagnostic and Statistical Manual of Mental Disorders (DSM), which included the use of the Autism Diagnostic Interview-Revised (ADI-R) and Autism Diagnostic Observation Schedule (ADOS).

**Table 1:** Participant's demographics: group, number of participants, ages, and IQ.

Group	Participants (females)	Age, years Mean(std)	IQ Mean(std)
ASD	15 (4)	10.8 (± 1.4)	95.7 (± 21)
Typically developing	37 (15)	9.3 (± 1.6)	109.6 (± 13.7)

### Data collection

**Figure 1:**
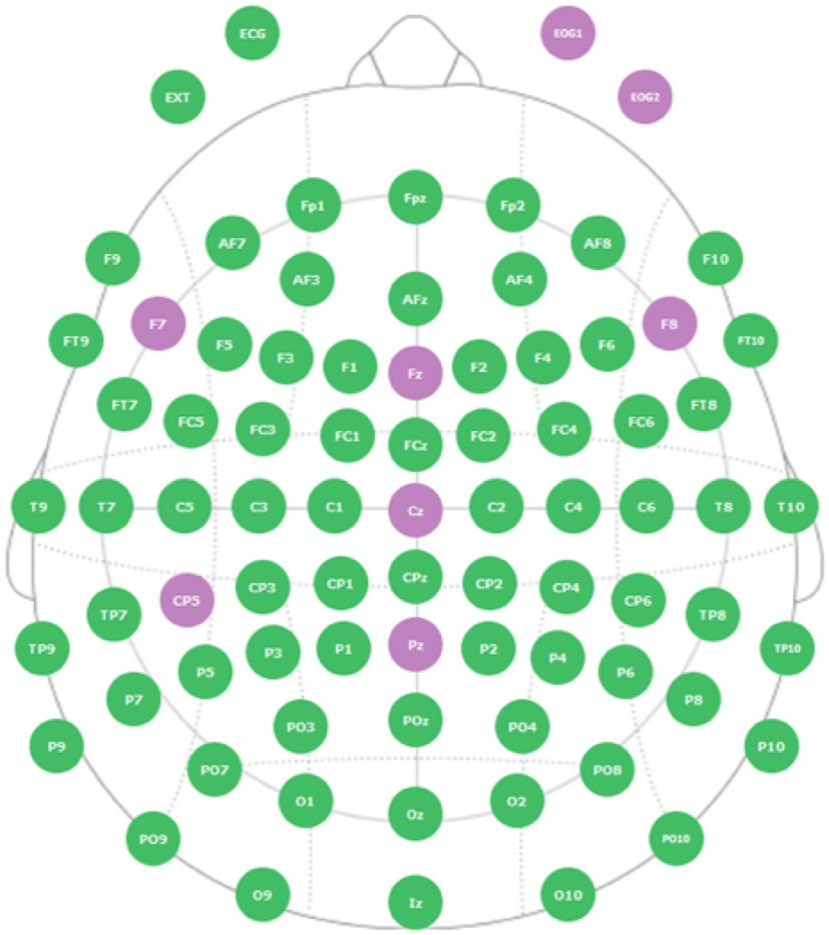
Locations of the eight recording electrodes (purple) with extended standard montage for reference (green).

Data were collected in parallel from multiple children during four single-day summer camps, across two years (2018–2019), using methods previously developed by our research group^74–80^. These summer camps involved multiple research groups running behavioural, and/or neurophysiological examinations on children with ASD and typically developing controls. EEG was recorded with ENOBIO systems (manufactured by Neuroelectrics) at the SFU's Behavioral and Cognitive Neuroscience Institute (BCNI). An ENOBIO system with a small number of EEG channels was chosen due to its comfort for the children, quick application, and reasonable signal quality^81^. Specifically, eight EEG channels were a priori selected as a compromise between a short preparation time, comfort for the children, and ability to cover more brain areas. The event-related potentials (ERP) were recorded with the sampling rate of 500 Hz from six electrodes: Fz, Cz, Pz, F7, F8, and CP5. Two electro-oculography (EOG) electrodes were placed above and beside the left eye (Fig. 1). The ground electrode was placed on the forehead, and the reference was placed on the right ear lobe. While recording EEG, we administered a language task with two conditions, with one condition having two sub-categories. Accuracy and reaction times were recorded in addition to electrophysiological data during task performance.

We performed our study was following the recommendations of the human research ethics guidelines from the Simon Fraser University (SFU) Office of Research Ethics. Written informed consent in accordance with the Declaration of Helsinki was obtained from each parent or guardian, and informed assent was obtained for each participant. The protocol was approved by the office of research ethics at SFU.

### Task conditions and stimuli

EEG was recorded during a computerized audio-visual task. Our experimental design included two task conditions. The first condition was 'congruent', wherein an image was presented with audio that accurately describes the image (33% of the total trials). The second condition was 'incongruent' wherein the image is presented with audio that describes it incorrectly. The 'incongruent condition' included two sub-categories: approximately half of the incongruent trials included a grammatically correct sentence (33% of the total trials), and the other half presented a sentence with semantic mistakes (33% of the total trials). The sentences used to describe the images were formed with two-word sentences using a verb and a monosyllabic noun.

The auditory descriptions and images were presented simultaneously, and the sound was delivered through headphones. The images appeared in the center of a computer screen at 0° (no text was included). The size of the images is 9.5 cm by 9.5 cm, and the approximate distance from the participant’s eyes to the monitor was 75 cm. Stimulus size in visual angle was equal to 7.6863. The images were selected from a comic book called 'MAFALDA' in black and white, which was created by Joaquín Salvador Lavado Tejón (1964–1973). Every image appeared three times, each time with a different description category. Participants were instructed to press one keyboard key with a green sticker if the image corresponded to the sentence they heard and one keyboard key with a red sticker if the image did not correspond to the sentence they heard.

**Figure 2:**
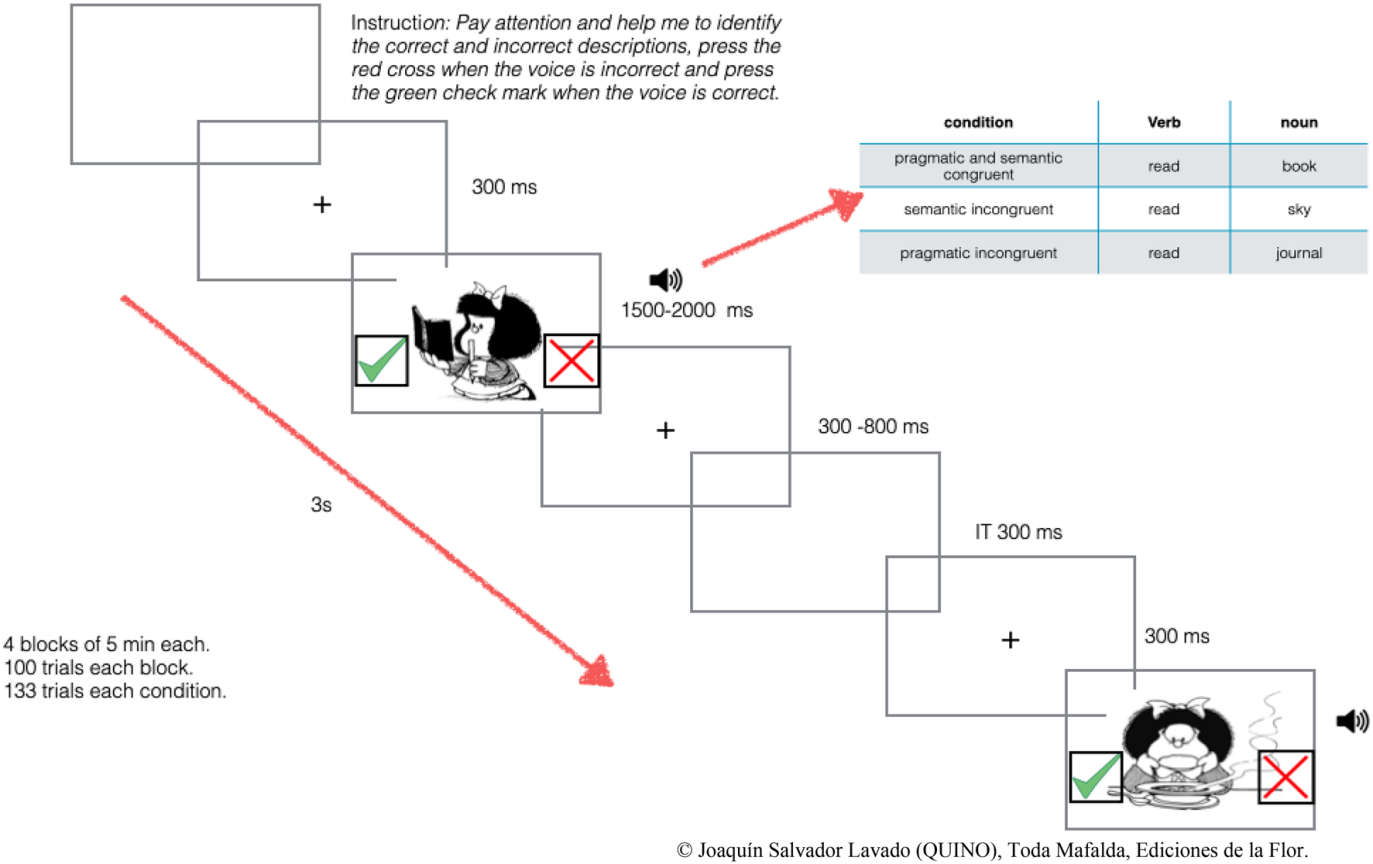
The stimulus display and its time course show the language task displaying one picture with the three possible conditions. After the presentation of the fixation cross, an image was presented simultaneously with the sound of a voice describing the image. The participants were required to press the green check mark (left keyboard arrow) if the description is correct and the red cross (right keyboard arrow) if it is incorrect.

To design the task, each image was presented to 20 typically developed, English-speaking adults. These adults were asked to describe the image in two words using one verb and one monosyllabic noun. The most common descriptions were considered for the congruent condition. To create the incongruent condition, we employed the help of an expert in linguistics. Once the sentences were selected, we showed the images and the incongruent sentences to the same 20 adults, and we found that the 20 typically developed adults were able to identify the congruent and incongruent descriptions with 100% accuracy. The audio was created using an online female voice generator (female robotic voice). The audio recordings were edited with the Audacity software and the length of the audio recordings was set between 1 and 2 s (Fig. 2).

### EEG pre-processing

EEG recordings were pre-processed in Matlab (MathWorks, version 9.7, R2019b), using the EEGLab toolbox, version 2019.0^82^. The signals recorded at lead EEG channels were band-pass filtered between 1 and 25 Hz. Channels with large artifacts were identified visually and removed from further analysis. Eye-movement artifacts were removed as described in a study comparing automatic methods for ocular artifact reduction in a similar situation with six lead EEG and two EOG channels^83^. The EOG signals were first band-pass filtered between 1 and 7 Hz, and then regressed out from the regular EEG signals, separately for each channel^83^. EEG data were detrended, and then epoched. To include the N400 and P600 components, we defined the trial as a time period between 200 ms before the onset of the auditory stimulus and 800 ms after the onset of the stimulus. We applied a baseline correction, with the epoch baseline defined as the first 200 ms of the trial ([− 200 0] ms).

Trials containing signals with median amplitudes greater than 150 μV or less than − 150 μV were removed from the analysis. Trials associated with the incorrect response or no response at all were also removed from analysis. Only subjects with more than 20 correct trials and less than 60 incorrect or with no response trials were included (Table 2). Channel-specific ERPs were computed by averaging the EEG signals across the trials. For the purpose of our study, the timing for the N400 and P600 components were defined a priori as advised in the literature^84^. Specifically, the timing for the N400 component was set between 200 and 500 ms with regard to the onset of the auditory stimulus. The interval of the P600 component was defined as 500 to 800 ms after the onset of the auditory stimulus (Table 3).

**Table 2:** Reports of accuracy in artifact-free trials: mean and standard deviation of the total of trials; mean and standard deviation of correct trials; percentage of correct trials mean and standard deviation; and response time for correct trials mean and standard deviation.

	Number of trials Mean (std)	Number of correct trials Mean (std)	Percentage of correct trials Mean (std)	Response times for correct trials Mean (std)
**ASD**				
Congruent	101 (± 31)	82 (± 27)	81 (± 7)	1.49 (± 0.46)
Incongruent (semantic)	103 (± 31)	82 (± 24)	80 (± 7)	1.75 (± 0.54)
Incongruent (pragmatic)	105 (± 29)	84 (± 25)	79 (± 9)	1.82 (± 0.72)
**Typically developing**				
Congruent	115 (± 30)	91 (± 27)	80 (± 11)	1.65 (± 0.58)
Incongruent (semantic)	117 (± 27)	94 (± 26)	81 (± 12)	1.64 (± 0.51)
Incongruent (pragmatic)	115 (± 30)	95 (± 28)	82 (± 11)	1.65 (± 0.48)

Response time was computed as a delay between the response and the end of the second word.

**Table 3:**
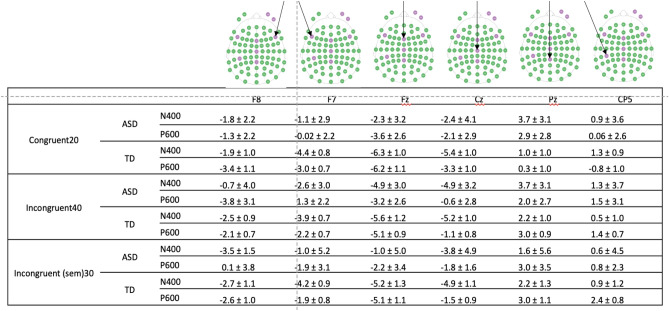
Average ERP in microvolts and standard error per channel, condition, component, and group.

### Multivariate statistical analysis of ERPs

To test for differences in ERPs between groups or conditions, we applied Partial Least Squares (PLS) analysis, a muti-variate approach wherein we can test all the time points and all the groups or conditions at once. The PLS approach is based on decomposing all data into a set of latent variables, similar to principal component analysis. PLS operates on the entire data structure at once with the data organized into matrices: subjects within groups or conditions times EEG features. In our analysis, EEG features were defined as the ERP signal amplitude for a specific range of data points, between 200 and 500 ms for the N400 component, or between 500 and 800 ms for the P600 component, with 150 data points or EEG features in both cases^85^.

Each latent variable from the data decomposition is associated with a vector representing a contrast across groups or conditions. The dimensionality of this vector is equal to the total number of experimental groups or conditions. For example, if we test for differences across conditions (congruent and incongruent, including semantic and pragmatic) in the ASD group, the dimensionality equals three. If we include two incongruent sub-categories such as pragmatic semantic, and two groups (ASD and TD), the dimensionality of this vector is equal to four. This vector can be interpreted as the overall differences between groups or conditions. It shows the difference between these groups or conditions 'on average': across all the EEG features included in the analysis. In our paper, we will call it the overall contrast or just the contrast (between groups or conditions).

As was originally adapted for neuroimaging studies, the PLS method typically includes a permutation test. The permutation test assesses the significance of the effect represented by the overall contrast by measuring how it is different from random noise. Such an approach alleviates the problem of multiple comparisons, as the permutation test generates one p-value for one contrast for all EEG features at once. Specifically, this test was performed using 1000 random permutations of subjects across the groups or/and conditions, estimating the significance of overall group/condition differences.

In our study, we used two types of PLS analyses: so-called Mean-Centered and Contrast PLS. Both approaches assess the significance of condition or group differences. The Mean-Centered PLS is a data-driven approach: the contrast is not specified a priori but rather determined by the variability in data themselves. Contrast PLS is an example of a modelling approach. With the Contrast PLS, the overall contrast is specified a priori. For example, this contrast is set as a two-dimensional vector in a scenario based on two groups and one condition:^1^. Regardless of the type of PLS analysis, one contrast is associated with one p-value for the entire ERP component.

### Statistical analysis of ERPs: differences across conditions

We applied the Mean-Centered PLS analysis to investigate differences in ERPs between conditions (one congruent and two incongruent conditions), separately for each group, for each electrode. The N400 and P600 components were tested separately. In total, we performed 24 PLS analyses: two groups (ASD and TD) times two ERP components (N400 and P600) times six electrodes. In each case, PLS returned a three-dimensional vector representing a data-driven, overall contrast across the three stimulus categories. Each contrast was associated with a p-value coming from the permutation test, and these p-values showed the significance of these differences.

### Statistical analysis of ERP: differences across groups

Mean-Centered PLS analysis was performed to investigate differences in ERPs between two groups (ASD and TD), separately for congruent and incongruent conditions and each electrode. The N400 and P600 components were tested separately. In total, we applied 24 PLS analyses: two conditions (congruent and incongruent) times two ERP components (N400 and P600) times six EEG electrodes. For the congruent condition, PLS returned a two-dimensional vector of overall group differences. For the incongruent conditions, PLS returned a four-dimensional data-driven contrast across the groups and two incongruent conditions. Each contrast was associated with a p-value based on the permutation test, and we determined the significance of this contrast based on this p-value.

## Results

### Atypical ERP responses in children with ASD

We performed a contrast PLS analysis, wherein a contrast between the two groups (ASD and typically developing) was tested. PLS was applied separately for each condition and each electrode. The group contrast was set as^1^ for the congruent condition and [1 1 − 1 1] for the incongruent conditions (two groups and two sub-categories at once). Table 4 summarizes the PLS results. Specifically, we observed statistically significant differences in P600 amplitudes between the two groups in the Fz and F8 electrodes for the congruent and incongruent conditions, and in the F7, Fz, and F8 electrodes for the incongruent condition. We also observed significant group differences in the N400 response in the incongruent conditions for the electrodes Cz and CP5.

**Table 4:** Differences between groups, in congruent and incongruent conditions on N400 and P600 components.

ERP	Condition	F7	Fz	F8	Cz	CP5	Pz
N400	Autistic vs typically developing congruent	0.85	0.71	0.28	0.68	0.44	0.54
	Autistic vs typically developing incongruent	0.19	0.16	0.15	**0.02**	**0.05**	0.30
P600	Autistic vs typically developing congruent	0.10	**0.02**	**0.01**	0.11	0.40	0.25
	autistic vs typically developing incongruent	**0.09**	**0.03**	**0.05**	0.74	0.63	0.14

Significant P values in bold.

**Figure 3:**
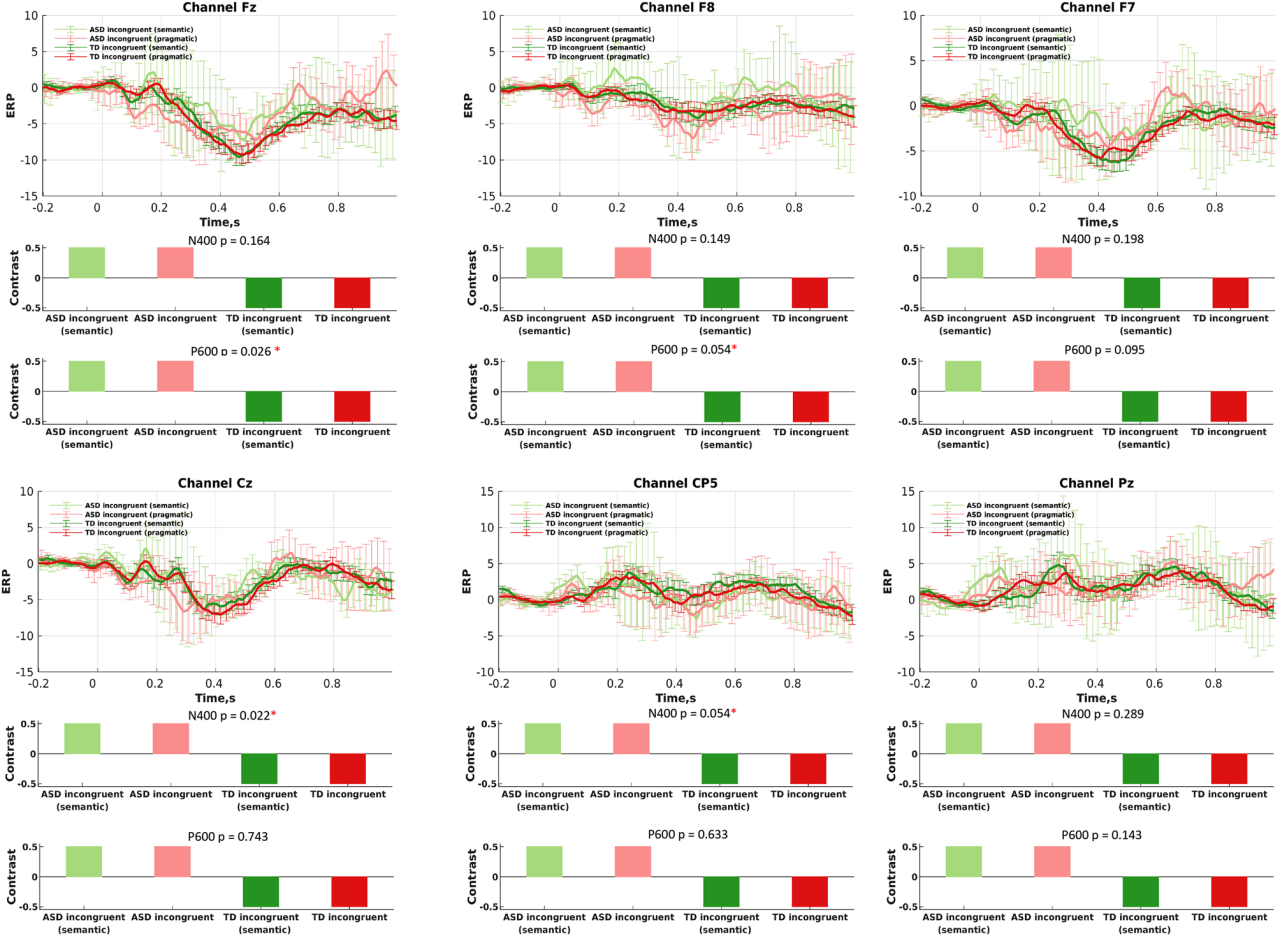
Differences in the N400 and P600 components between incongruent conditions performed by the autistic and non-autistic groups: (upper sub-plots) ERPs (condition means); and (lower sub-plots) data-driven contrasts between these four experimental conditions, as revealed by mean-centered PLS analysis.

**Figure 4:**
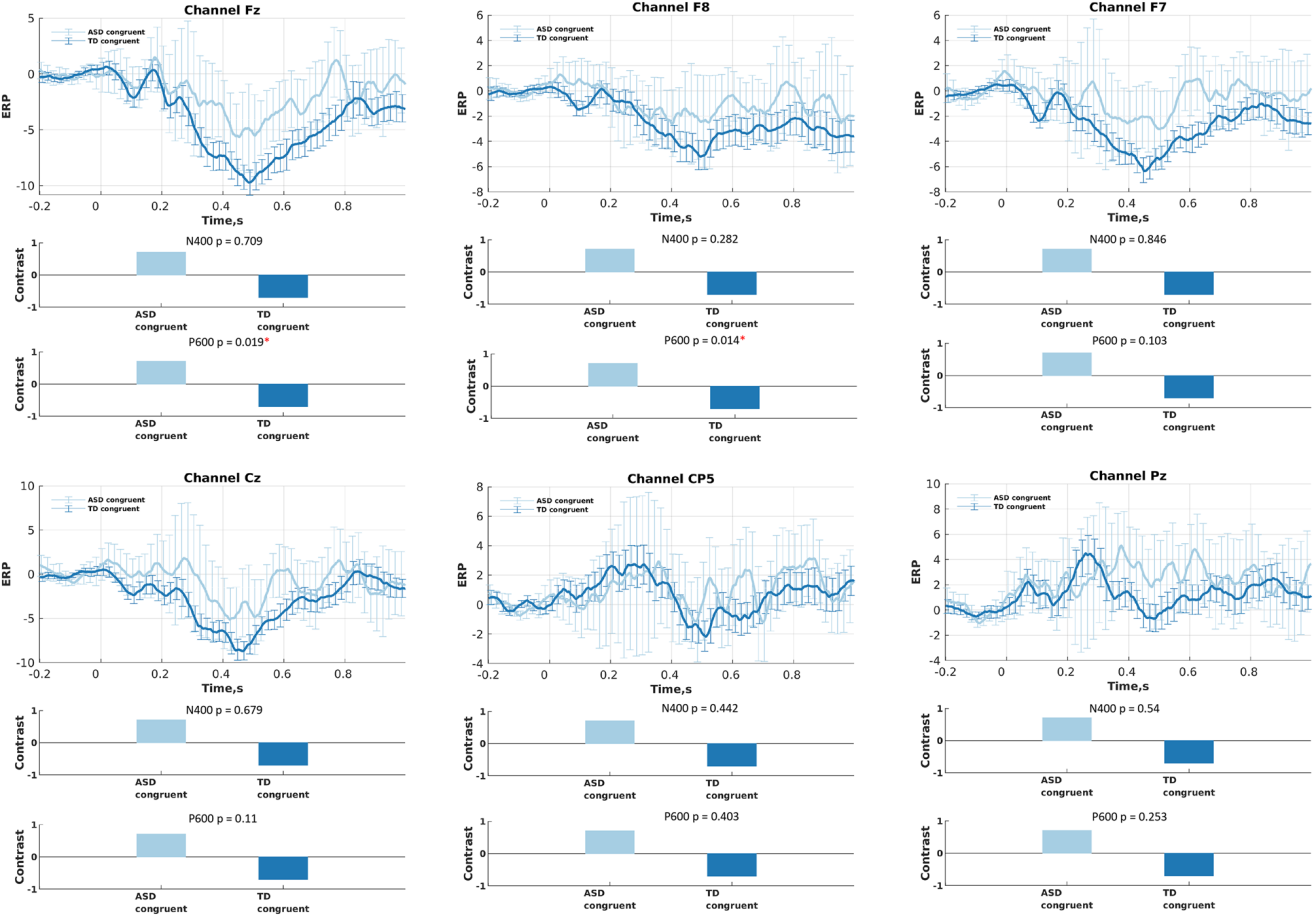
Differences in the N400 and P600 components between congruent conditions performed by the autistic and non-autistic groups: (upper sub-plots) ERPs (condition means); and (lower sub-plots) data-driven contrasts between these two experimental conditions, as revealed by mean-centered PLS analysis.

Figure 3 shows all the group-averaged ERPs for the incongruent condition, separately for each electrode, as well as the a priori selected contrast tested with Contrast PLS analysis. Similar to Fig. 3, Fig. 4 shows all the group-averaged ERPs for the congruent language condition.

### ERP differences between congruent and incongruent conditions

**Figure 5:**
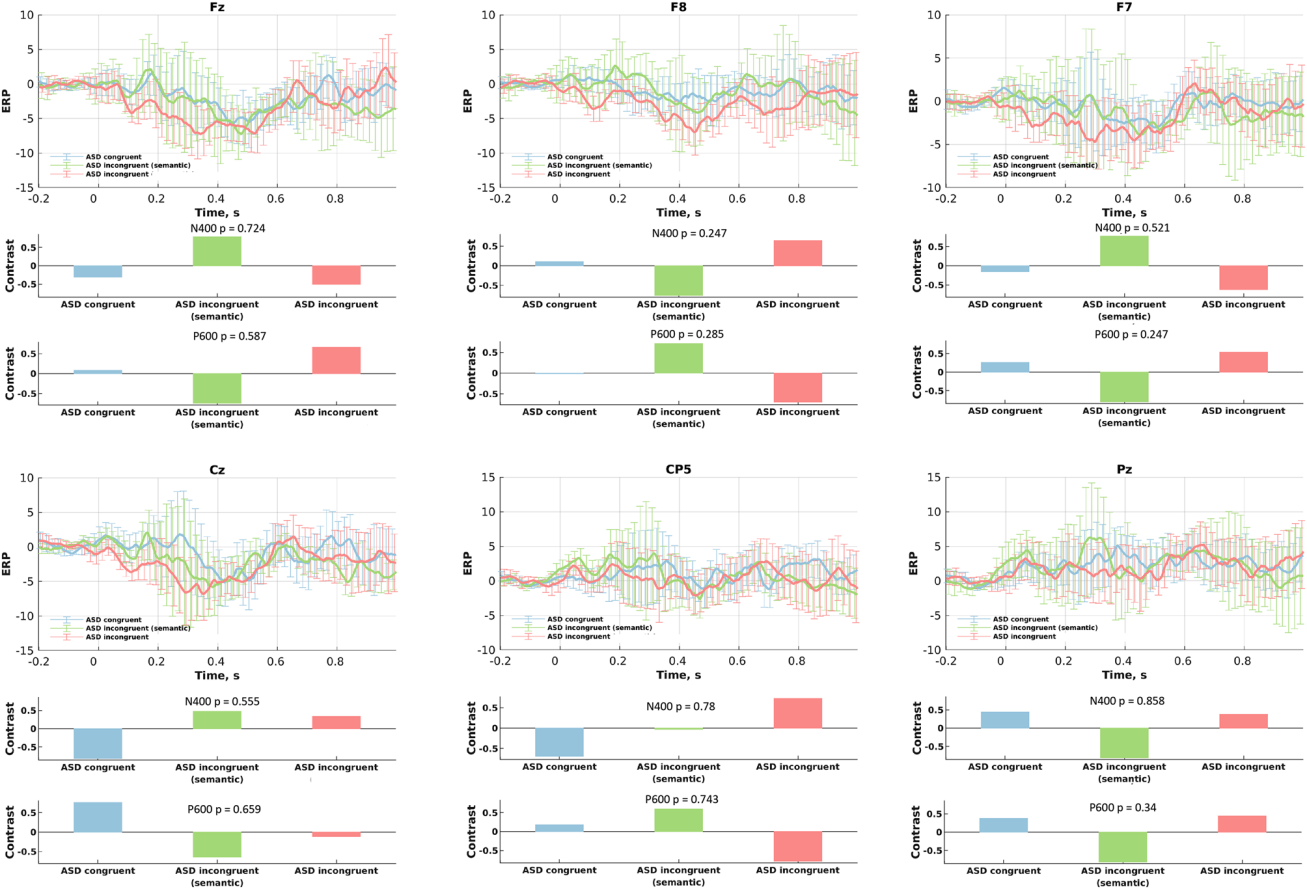
Differences in the N400 and P600 components between three conditions performed by the autistic cohort: (upper sub-plots) ERPs (condition means); and (lower sub-plots) data-driven contrasts between these three experimental conditions, as revealed by the Mean-Centered PLS analysis.

**Figure 6:**
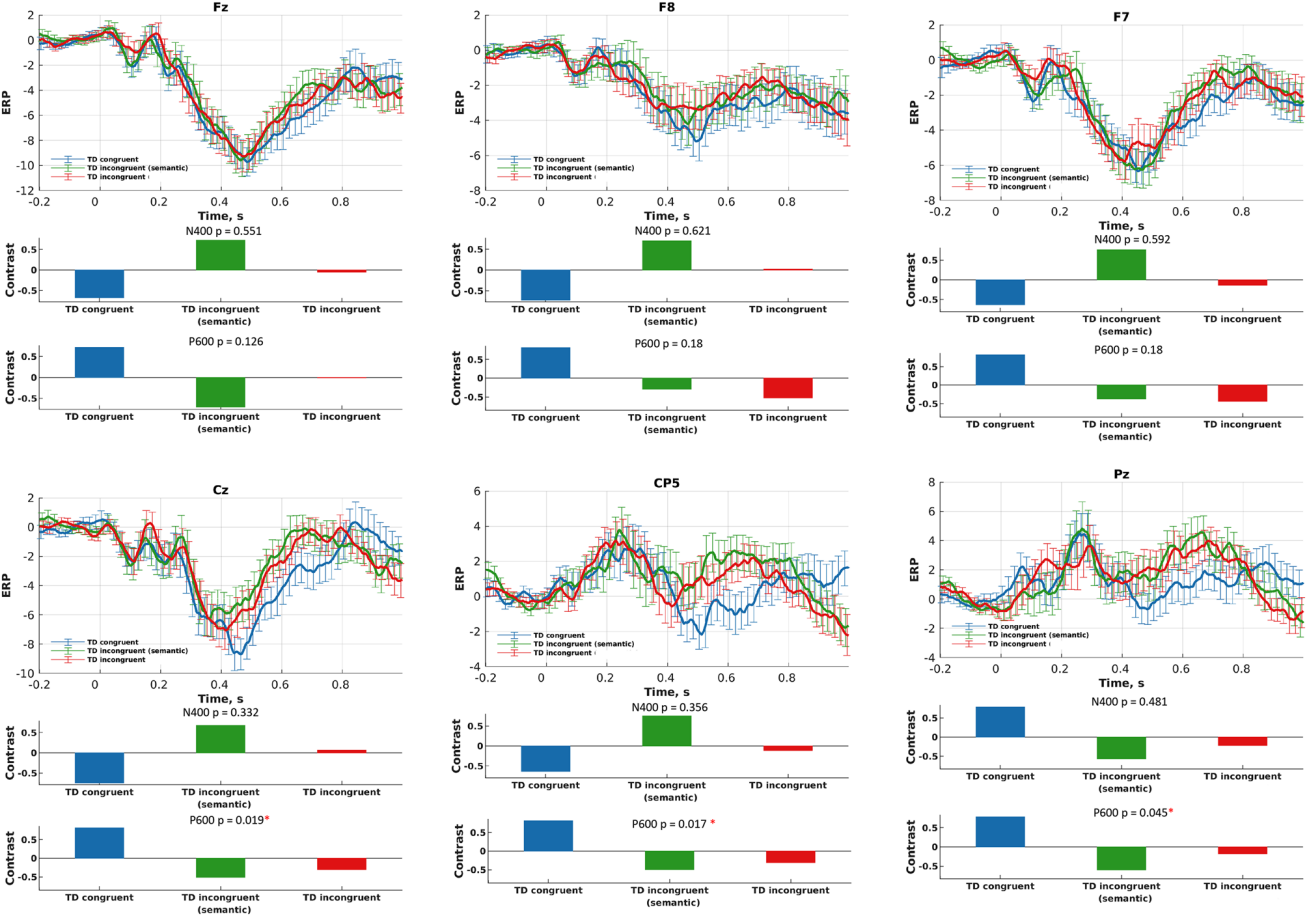
Differences in the N400 and P600 components between three language conditions performed by the non-autistic cohort: (upper sub-plots) ERPs (condition means); and (lower sub-plots) data-driven contrasts between these three experimental conditions, as revealed by mean-centered PLS analysis. Note that the differences in ERP across the three conditions are significant only for the P600 component for electrodes Cz, CP5, and Pz.

The ASD group presented no statistically significant differences between conditions in both N400 and P600 for all the electrodes (p-values were between 0.24 and 0.85) (Fig. 5). No significant differences were detected for N400 across conditions in the typically developing group (p values between 0.33 and 0.62), as illustrated in Fig. 6. However, the PLS analysis revealed a contrast in the ERPs between conditions for the P600 component in the typically developing group, which was significant at the 95% confidence interval for the electrodes Cz (p = 0.019) and CP5 (p = 0.017), and Pz (p = 0.045). Figure 6 also shows the results from mean-centered PLS analysis: specifically, the data-driven contrasts in the N400 or P600 component between the three experimental conditions and the corresponding ERPs for the TD group, separately for each electrode. Note that for the significant results (P600 for electrodes Cz, CP5, and Pz), the three-dimensional contrasts represent differences between the congruent and the two incongruent conditions separately.

## Discussion

Difficulties with social communication is a defining feature of ASD, even in fluently verbal individuals^8,9^. Partly, these challenges arise from the difficulty in integrating contextual clues, which usually aid in accurately interpreting messages and intentions. The present study compared the electrophysiological response in language comprehension in autistic and typically developing children. Specifically, we investigated the amplitude on the N400 and P600 ERP components during a language processing task that included context congruent and incongruent conditions.

Here we propose a new experimental paradigm to investigate the social communication difficulties in autism. The results are consistent with the previously reported difficulties in the processing of language in context, in participants with ASD. These cognitive differences were also accompanied by neurophysiological markers as shown by the electrophysiological recordings. We found children with ASD showed similar electrophysiological responses in both conditions (congruent and incongruent), whereas, the typically developing group showed differences in their electrophysiological responses (ERP amplitudes) to both conditions. In addition, the two groups presented significant differences in the amplitudes of the ERP components, suggesting difficulties detecting contextual incongruencies in the autistic group.

### P600 component

The P600 component is commonly associated with syntactic anomalies such as a number mismatch between the elements of a sentence^57,63^, violations of gender agreement^61^, tense inflection^86^, and violations of phrase structure^57,87^. It has also been described as the result of integration among different information streams relatively early in the comprehension of an utterance^88^. Furthermore, P600 has been recognized as the result oflearning processes, rather than the processes underlying the comprehension of meaning^68^. Previous studies involving ASD populations have shown differences in the P600 amplitudes and reaction times when exploring linguistic violations^72,73^. In our study, these differences were tested not only with linguistic violations but with the processing of contextual information in an audiovisual task. Interestingly, our results show differences between groups in both the congruent and the incongruent conditions. Differences in the P600 amplitudes between groups in both congruent and incongruent conditions can be associated with difficulties integrating both sources of information (auditory and visual). This result suggests that autistic children may not readily integrate new contextual information when processing a multi-modal task.

In addition, our results showed that the ASD group presented no significant differences between congruent and incongruent conditions as indicated by the P600 amplitude. These results suggest that children with ASD show no neurophysiological evidence of detecting the incongruencies, contributing to a better understanding of the difficulties in comprehension of language in context in ASD. In comparison, the typically developing group presented statistically significant differences in the P600 amplitudes between conditions. These differences show that this task is capable of eliciting electrophysiological differences between conditions and serves as a control contrast between groups.

### N400 component

The N400 is the most widely used ERP in language research^27^. Reduced N400 amplitudes have been previously observed in children and adults with ASD when responding to auditory sentences, in comparison with control groups^50,52,89,90^. These results have been interpreted as suggesting that individuals with ASD make less use of contextual information, which could be due to a less elaborate or less connected semantic network.

Our study evaluated the differences between groups for the N400 amplitudes, and in the incongruent conditions, the groups presented significant differences. However, neither group expressed significant differences across conditions. Such results are in line with previous findings on restricted pragmatic and semantic processing of verbal sentences in the ASD population^50,52,55,89,91–94^.

On the other hand, when evaluating the differences between groups for the N400 amplitudes in the congruent condition, the groups did not present significant differences. These results were expected considering that the N400 commonly does not appear under congruent conditions and this condition was intended to be the control condition. These results, however, could also suggest that children with ASD do not have difficulty accessing semantic memory (N400) for the auditory and visual task, when the information is congruent or expected^95^.

Overall, our study suggests that children with ASD may have more difficulty than controls in processing contextual information. By analyzing the amplitudes of N400 and P600 components, we found differences in neurophysiological responses related to the integration of contextual information in autistic children compared to non-autistic children. These results are consistent with the difficulties that individuals with ASD often have detecting incongruencies between language and context. Further, this study suggests that N400 and P600 amplitudes, used in the detection of incongruencies between images and its description, are a sensitive marker of differences in language processing between children with and without ASD.

### Correct trials and reaction times

Previous studies have suggested differences in behavioral performance (i.e. accurately differentiating the congruent and incongruent conditions) and reaction times between typically-developing children and children with ASD^96^. However, our study found no significant differences between groups on these two measures. Interestingly, even when both groups have similar behavioral responses, we observed significant differences on the electrophysiological responses. Speculatively, the observed differences in N400 and P600 could provide evidence of a compensatory effect that ultimately is effective at facilitating typical behavioral performance and contributing to a typical adequate reaction time. Our results indicate the need to further explore neurophysiological mechanisms underlying the pragmatic language processing differences in ASD.

### Limitations

More research is needed on pragmatic language and its relationship with social communication challenges in ASD. Studies using neuroimaging modalities with a higher number of autistic individuals of both genders and at various levels of functioning would be ideal. In addition, longitudinal studies, or studies with a greater range of ages could better explore the development and change in social communication skills over time and the optimal developmental periods for interventions. Increasing the number of channels used in the EEG could open the possibility of exploring the topology of the ERPs. It is also important to note that the current experimental setting might differ significantly from a natural situation of social communication.

Finally a potential confound that needs to be addressed in the study of pragmatic language communication is the issue of multisensory integration in autistic individuals, which explains that these individuals exhibit alterations in sensory processing, including changes in the integration of information across the different sensory modalities^97^. Despite the limitations, this study provides important new findings that show significant differences between autistic and non-autistic children in the amplitudes of the N400 and P600 components during a pragmatic language task.

## References

[1] Frith U (1989). A new look at language and communication in autism. Int. J. Lang. Commun. Disord..

[2] Frith, U. *Autism: Explaining the Enigma*, 2nd edn. (Blackwell Publ., 2003), Accessed Feb 27, 2021. [https://psycnet.apa.org/record/2003-00578-000.

[3] American Psychiatric Association. *Diagnostic and Statistical Manual of Mental Disorders (DSM-5®)*. (2013).

[4] Baixauli Fortea I, Berenguer Forner C, Colomer C, Casas AM, Miranda BR (2018). Communicative skills in Spanish children with Autism Spectrum Disorder and children with Attention Deficit Hyperactivity Disorder. Analysis through parents’ perceptions and narrative production. Res. Autism Spectr. Disord..

[5] Klusek J, Martin GE, Losh M (2013). Physiological arousal in autism and fragile X syndrome: Group comparisons and links with pragmatic language. Am. J. Intellect. Dev. Disabil..

[6] Larkin F, Hobson JA, Hobson RP, Tolmie A (2017). Collaborative competence in dialogue: Pragmatic language impairment as a window onto the psychopathology of autism. Res. Autism Spectr. Disord..

[7] Martin GE, Bush L, Klusek J, Patel S, Losh M (2018). A multimethod analysis of pragmatic skills in children and adolescents with fragile X syndrome, autism spectrum disorder, and down syndrome. J. Speech Lang. Hear. Res..

[8] Happé FGE (1997). Central coherence and theory of mind in autism: Reading homographs in context. Br. J. Dev. Psychol..

[9] Jolliffe T, Baron-Cohen S (1999). A test of central coherence theory: Linguistic processing in high-functioning adults with autism or Asperger syndrome: Is local coherence impaired?. Cognition.

[10] Lönnqvist L (2017). How young adults with autism spectrum disorder watch and interpret pragmatically complex scenes. Q. J. Exp. Psychol..

[11] Barstein J, Martin GE, Lee M, Losh M (2018). A duck wearing boots?! pragmatic language strategies for repairing communication breakdowns across genetically based neurodevelopmental disabilities. J. Speech Lang. Hear. Res..

[12] Barzy M, Filik R, Williams D, Ferguson HJ (2020). Emotional Processing of Ironic Versus Literal Criticism in Autistic and Nonautistic Adults: Evidence From Eye-Tracking. Autism Res..

[13] Ben-Yosef D, Anaki D, Golan O (2017). Context processing in adolescents with autism spectrum disorder: How complex could it be?. Autism Res..

[14] Berenguer C, Miranda A, Colomer C, Baixauli I, Roselló B (2018). Contribution of theory of mind, executive functioning, and pragmatics to socialization behaviors of children with high-functioning autism. J. Autism Dev. Disord..

[15] De Marchena A, Eigsti IM (2016). The art of common ground: Emergence of a complex pragmatic language skill in adolescents with autism spectrum disorders. J. Child Lang..

[16] Deliens G, Papastamou F, Ruytenbeek N, Geelhand P, Kissine M (2018). Selective pragmatic impairment in autism spectrum disorder: Indirect requests versus irony. J. Autism Dev. Disord..

[17] Helland WA, Helland T (2017). Emotional and behavioural needs in children with specific language impairment and in children with autism spectrum disorder: The importance of pragmatic language impairment. Res. Dev. Disabil..

[18] Ihsan KB, Rizky YN (2018). Pragmatic and conversational features of arabic-speaking adolescents with autism spectrum disorder: Examining performance and caregivers’ perceptions. Arch. Anesthesiol. Crit. Care.

[19] Jyotishi M, Fein D, Naigles L (2017). Investigating the grammatical and pragmatic origins of wh-questions in children with autism spectrum disorders. Front. Psychol..

[20] Miller M, Young GS, Hutman T, Johnson S, Schwichtenberg AJ, Ozonoff S (2015). Early pragmatic language difficulties in siblings of children with autism: Implications for DSM-5 social communication disorder?. J. Child Psychol. Psychiatry Allied Discip..

[21] Miniscalco C, Rudling M, Gillberg C, Johnels JÅ (2014). Imitation (rather than core language) predicts pragmatic development in young children with ASD: A preliminary longitudinal study using CDI parental reports. Int. J. Lang. Commun. Disord..

[22] Rosello B, Berenguer C, Baixauli I, García R, Miranda A (2020). Theory of mind profiles in children with autism spectrum disorder: Adaptive/social skills and pragmatic competence. Front. Psychol..

[23] Feldman JI, Dunham K, Cassidy M, Wallace MT, Liu Y, Woynaroski TG (2018). Audiovisual multisensory integration in individuals with autism spectrum disorder: A systematic review and meta-analysis. Neurosci. Biobehav. Rev..

[24] Tager-Flusberg H, Kasari C (2013). Minimally verbal school-aged children with autism spectrum disorder: The neglected end of the spectrum. Autism Res..

[25] Bedo N, Ribary U, Ward LM (2014). Fast dynamics of cortical functional and effective connectivity during word reading. PLoS ONE.

[26] Kutas M, Federmeier KD (2011). Thirty years and counting: Finding meaning in the N400 component of the event-related brain potential (ERP). Annu. Rev. Psychol..

[27] Leckey M, Federmeier KD, Leckey M, Federmeier KD (2019). Electrophysiological methods in the study of language processing. The Oxford Handbook of Neurolinguistics.

[28] Leckey M, Federmeier KD (2020). The P3b and P600(s): Positive contributions to language comprehension. Psychophysiology.

[29] Kutas M, Van Petten C (1988). Event-related brain potential studies of language. Adv. Psychophysiol.

[30] Kutas M, Van Petten CK, Kluender R (2006). Psycholinguistics Electrified II (1994–2005). Handbook of Psycholinguistics.

[31] Holcomb PJ, Neville HJ (1990). Auditory and visual semantic priming in lexical decision: A comparison using event-related brain potentials. Lang. Cogn. Process..

[32] Holcomb PJ, Kounios J, Anderson JE, West WC (1999). Dual-coding, context-availability, and concreteness effects in sentence comprehension: An electrophysiological investigation. J. Exp. Psychol. Learn. Mem. Cogn..

[33] Herbert C, Kissler J (2014). Event-related potentials reveal task-dependence and inter-individual differences in negation processing during silent listening and explicit truth-value evaluation. Neuroscience.

[34] Wong W, Wu Y, Chen V (2014). Limited role of phonology in reading chinese two-character compounds: Evidence from an ERP study. Neuroscience.

[35] Holcomb PJ, Anderson JE (1993). Cross-modal semantic priming: A time-course analysis using event-related brain potentials. Lang. Cogn. Process..

[36] Orgs G, Lange K, Dombrowski J, Heil M (2006). Conceptual priming for environmental sounds and words: An ERP study. Brain Cogn..

[37] Senkowski D, Saint-amour D, Kelly SP, Foxe JJ (2007). Multisensory processing of naturalistic objects in motion : A high-density electrical mapping and source estimation study. Hum. Brain Mapp. J..

[38] Schneider TR, Debener S, Oostenveld R, Engel AK (2008). Enhanced EEG gamma-band activity reflects multisensory semantic matching in visual-to-auditory object priming. NeuroImage.

[39] Liu B, Wu G, Wang Z, Ji X (2011). Neuroscience Letters Semantic integration of differently asynchronous audio–visual information in videos of real-world events in cognitive processing : An ERP study. Neurosci. Lett..

[40] Liu B, Wu G, Meng X, Dang J (2013). Correlation between prime duration and semantic priming effect: Evidence from N400 effect. Neuroscience.

[41] Wu Z, Cao Z (2005). Improved MFCC-based feature for robust speaker identification. Tsinghua Sci. Technol..

[42] Halgren E (2002). N400-like magnetoencephalography responses modulated by semantic context, word frequency, and lexical class in sentences. Neuroimage.

[43] Helenius P, Salmelin R, Service E, Connolly JF (1998). Distinct time courses of word and context comprehension in the left temporal cortex. Brain.

[44] Helenius P, Salmelin R, Service E, Connolly JF, Leinonen S, Lyytinen H (2002). Cortical activation during spoken-word segmentation in nonreading-impaired and dyslexic adults. J. Neurosci..

[45] Kwon H, Kuriki S, Kim JM, Lee YH, Kim K, Nam K (2005). MEG study on neural activities associated with syntactic and semantic violations in spoken Korean sentences. Neurosci. Res..

[46] Simos PG, Basile LFH, Papanicolaou AC (1997). Source localization of the N400 response in a sentence-reading paradigm using evoked magnetic fields and magnetic resonance imaging. Brain Res..

[47] Coulson S, King JW, Kutas M (1998). Expect the unexpected: Event-related brain response to morphosyntactic violations. Lang. Cogn. Process..

[48] Coderre EL, Chernenok M, Gordon B, Ledoux K (2017). Linguistic and non-linguistic semantic processing in individuals with autism spectrum disorders: An ERP study. J. Autism Dev. Disord..

[49] DiStefano C, Senturk D, Jeste SS (2019). ERP evidence of semantic processing in children with ASD. Dev. Cogn. Neurosci..

[50] Fishman I, Yam A, Bellugi U, Lincoln A, Mills D (2011). Contrasting patterns of language-associated brain activity in autism and Williams syndrome. Soc. Cogn. Affect. Neurosci..

[51] Méndez M, Sans O, Abril B, Valdizan JR (2009). Event-related potentials (N 400) in autistic children. Clin. Neurophysiol..

[52] Dunn MA, Bates JC (2005). Developmental Change in Neutral Processing of Words by Children with Autism. J. Autism Dev. Disord..

[53] McCleery JP, Ceponiene R, Burner KM, Townsend J, Kinnear M, Schreibman L (2010). Neural correlates of verbal and nonverbal semantic integration in children with autism spectrum disorders. J. Child Psychol. Psychiatry Allied Discip..

[54] Ribeiro TC, Valasek CA, Minati L, Boggio PS (2013). Altered semantic integration in autism beyond language: A cross-modal event-related potentials study. NeuroReport.

[55] Pijnacker J, Geurts B, van Lambalgen M, Buitelaar J, Hagoort P (2010). Exceptions and anomalies: An ERP study on context sensitivity in autism. Neuropsychologia.

[56] Kuhl PK, Coffey-Corina S, Padden D, Dawson G (2005). Links between social and linguistic processing of speech in preschool children with autism: Behavioral and electrophysiological measures. Dev. Sci..

[57] Hagoort P, Brown C, Groothusen J (1993). The syntactic positive shift (sps) as an erp measure of syntactic processing. Lang. Cogn. Process..

[58] Bornkessel-schlesewsky I, Schlesewsky M (2008). An alternative perspective on ‘ semantic P600 ’ effects in language comprehension. Brain Res. Rev..

[59] Kuperberg GR (2007). Neural mechanisms of language comprehension: Challenges to syntax. Brain Res..

[60] Van Petten C, Luka BJ (2012). Prediction during language comprehension: Benefits, costs, and ERP components. Int. J. Psychophysiol..

[61] Osterhout L, Holcomb PJ (1993). Event-related potentials and syntactic anomaly: Evidence of anomaly detection during the perception of continuous speech. Lang. Cogn. Process..

[62] Ryskin R, Levy RP, Fedorenko E (2020). Do domain-general executive resources play a role in linguistic prediction? Re-evaluation of the evidence and a path forward. Neuropsychologia.

[63] Hagoort P, Brown CM (2000). ERP effects of listening to speech compared to reading: The P600/SPS to syntactic violations in spoken sentences and rapid serial visual presentation. Neuropsychologia.

[64] Gunter TC, Stowe LA, Mulder G (1997). When syntax meets semantics. Psychophysiology.

[65] Neville H, Nicol JL, Barss A, Forster KI, Garrett MF (1991). Syntactically based sentence processing classes: Evidence from event-related brain potentials. J. Cogn. Neurosci..

[66] Leckey M, Federmeier KD (2017). Age-related shifts in hemispheric dominance for syntactic processing. Psychophysiology.

[67] Tanner D, Van Hell JG (2014). ERPs reveal individual differences in morphosyntactic processing. Neuropsychologia.

[68] Fitz H, Chang F (2019). Language ERPs reflect learning through prediction error propagation. Cogn. Psychol..

[69] Sassenhagen J, Schlesewsky M, Bornkessel-Schlesewsky I (2014). The P600-as-P3 hypothesis revisited: Single-trial analyses reveal that the late EEG positivity following linguistically deviant material is reaction time aligned. Brain Lang..

[70] Fitz H, Chang F (2019). Language ERPs reflect learning through prediction error propagation. Cogn. Psychol..

[71] Sitnikova T, Holcomb PJ, Kuperberg GR (2008). Neurocognitive mechanisms of human comprehension. Understanding Events: From Perception to Action.

[72] Koolen S, Vissers CTWM, Hendriks AWCJ, Egger JIM, Verhoeven L (2012). The interplay between attentional strategies and language processing in high-functioning adults with autism spectrum disorder. J. Autism Dev. Disord..

[73] Koolen S, Vissers CTWM, Egger JIM, Verhoeven L (2014). Monitoring in language perception in high-functioning adults with autism spectrum disorder: Evidence from event-related potentials. Clin. Neurophysiol..

[74] Fong VC, Iarocci G (2020). The role of executive functioning in predicting social competence in children with and without autism spectrum disorder. Autism Res..

[75] Hutchison SM, Müller U, Iarocci G (2020). Parent reports of executive function associated with functional communication and conversational skills among school age children with and without autism spectrum disorder. J. Autism Dev. Disord..

[76] Magnuson JR (2019). Electrophysiology of inhibitory control in the context of emotion processing in children with autism spectrum disorder. Front. Hum. Neurosci..

[77] Magnuson JR, Iarocci G, Doesburg SM, Moreno S (2020). Increased intra-subject variability of reaction times and single-trial event-related potential components in children with autism spectrum disorder. Autism Res..

[78] Moreno S, Bialystok E, Barac R, Schellenberg EG, Cepeda NJ, Chau T (2011). Short-term music training enhances verbal intelligence and executive function. Psychol. Sci..

[79] Moreno S, Lee Y, Janus M, Bialystok E (2015). Short-term second language and music training induces lasting functional brain changes in early childhood. Child Dev..

[80] Moreno S, Bidelman GM (2014). Examining neural plasticity and cognitive benefit through the unique lens of musical training. Hearing Res..

[81] Cimtay Y, Ekmekcioglu E (2020). Investigating the use of pretrained convolutional neural network on cross-subject and cross-dataset eeg emotion recognition. Sensors.

[82] Delorme A, Makeig S (2004). EEGLAB: An open source toolbox for analysis of single-trial EEG dynamics. J. Neurosci. Methods.

[83] Romero S, Mañanas MA, Barbanoj MJ (2008). A comparative study of automatic techniques for ocular artifact reduction in spontaneous EEG signals based on clinical target variables: A simulation case. Comput. Biol. Med..

[84] De Zubicaray GI, Schiller NO (2019). The Oxford Handbook of Neurolinguistics.

[85] Krishnan A, Williams LJ, McIntosh AR, Abdi H (2011). Partial Least Squares (PLS) methods for neuroimaging: A tutorial and review. Neuroimage.

[86] Allen M, Badecker W, Osterhout L (2003). Morphological analysis in sentence processing: An ERP study. Lang. Cogn. Process..

[87] Friederici AD, Hahne A, Mecklinger A (1996). Temporal structure of syntactic parsing. J. Exp. Psychol. Learn. Mem. Cogn..

[88] Spotorno N, Cheylus A, Van Der Henst JB, Noveck IA (2013). What’s behind a P600? Integration operations during irony processing. PLoS ONE.

[89] Dunn M, Vaughan H, Kreuzer J, Kurtzberg D (1999). Electrophysiologic Correlates of Semantic Classification in Autistic and Normal Children. Dev. Neuropsychol..

[90] O’connor K (2012). Auditory processing in autism spectrum disorder: A review. Neurosci. Biobehav. Rev..

[91] Braeutigam S, Swithenby SJ, Bailey AJ (2008). Contextual integration the unusual way: A magnetoencephalographic study of responses to semantic violation in individuals with autism spectrum disorders. Eur. J. Neurosci..

[92] Coderre EL, Cohn N, Slipher SK, Chernenok M, Ledoux K, Gordon B (2018). Visual and linguistic narrative comprehension in autism spectrum disorders: Neural evidence for modality-independent impairments. Brain Lang..

[93] Kujala T, Lepistö T, Näätänen R (2013). The neural basis of aberrant speech and audition in autism spectrum disorders. Neurosci. Biobehav. Rev..

[94] Lepistö T, Kujala T, Vanhala R, Alku P, Huotilainen M, Näätänen R (2005). The discrimination of and orienting to speech and non-speech sounds in children with autism. Brain Res..

[95] Manfredi M, Cohn N, Sanchez Mello P, Fernandez E, Boggio PS (2020). Visual and verbal narrative comprehension in children and adolescents with autism spectrum disorders: An ERP study. J. Autism Dev. Disord..

[96] Schmitz N, Daly E, Murphy D (2007). Frontal anatomy and reaction time in Autism. Neurosci. Lett..

[97] Stevenson RA (2014). Evidence for diminished multisensory integration in autism spectrum disorders. J. Autism Dev. Disord..

